# Three lateral osteotomy designs for bilateral sagittal split osteotomy: biomechanical evaluation with three-dimensional finite element analysis

**DOI:** 10.1186/1746-160X-6-4

**Published:** 2010-03-26

**Authors:** Hiromasa Takahashi, Shigeaki Moriyama, Haruhiko Furuta, Hisao Matsunaga, Yuki Sakamoto, Toshihiro Kikuta

**Affiliations:** 1 Department of Oral and Maxillofacial Surgery, Faculty of Medicine, Fukuoka University, 7-45-1 Nanakuma, Jonan-ku, Fukuoka, Japan; 2 Department of Mechanical Engineering, Faculty of Engineering, Fukuoka University, 8-19-1 Nanakuma, Jonan-ku, Fukuoka, Japan

## Abstract

**Background:**

The location of the lateral osteotomy cut during bilateral sagittal split osteotomy (BSSO) varies according to the surgeon's preference, and no consensus has been reached regarding the ideal location from the perspective of biomechanics. The purpose of this study was to evaluate the mechanical behavior of the mandible and screw-miniplate system among three lateral osteotomy designs for BSSO by using three-dimensional (3-D) finite element analysis (FEA).

**Methods:**

The Trauner-Obwegeser (TO), Obwegeser (Ob), and Obwegeser-Dal Pont (OD) methods were used for BSSO. In all the FEA simulations, the distal segments were advanced by 5 mm. Each model was fixed by using miniplates. These were applied at four different locations, including along Champy's lines, to give 12 different FEA miniplate fixation methods. We examined these models under two different loads.

**Results:**

The magnitudes of tooth displacement, the maximum bone stress in the vicinity of the screws, and the maximum stress on the screw-miniplate system were less in the OD method than in the Ob and TO methods at all the miniplate locations. In addition, Champy's lines models were less than those at the other miniplate locations.

**Conclusions:**

The OD method allows greater mechanical stability of the mandible than the other two techniques. Further, miniplates placed along Champy's lines provide greater mechanical advantage than those placed at other locations.

## Background

Bilateral sagittal split osteotomy (BSSO) is the most common orthognathic surgical procedure [1]. It was first described by Trauner and Obwegeser in 1957 [2]. Since then, several modifications of the technique have been introduced with the aim of improving surgical convenience, minimizing morbidity, and maximizing procedural stability. These modifications include the technique described by Dal Pont [3]; it is generally recognized that the buccal osteotomy cut of the Obwegeser-Dal Pont method is positioned more anteriorly than that of the Obwegeser method [4], thereby increasing the amount of cancellous bone contact.

There are several factors determining the optimal modification for BSSO in a patient, including the position of the mandibular foramen (lingual), course of the inferior alveolar nerve in the mandible, presence of the mandibular third molars, and planned direction and magnitude of distal segment movement [5]. However, the location of the lateral osteotomy cut for BSSO varies according to the surgeon's preference, and no consensus has been reached regarding the ideal location from the perspective of biomechanics. Although biomechanics is only one of the factors determining the osteotomy technique to be used, it is important for the surgeon to consider the presence of jaw deformities while planning the treatment strategy.

Rigid internal fixation is routinely used to stabilize the proximal and distal segments following BSSO, for fast bone healing, initiating early postoperative mandibular function, and decreasing the amount of relapse [6]. Similarly, a stable osteotomy design is desired. Although numerous studies have been conducted to compare the different types of fixation techniques, experiments comparing different BSSO techniques for use in orthognathic surgery are limited [7].

Korkmaz et al. [8] have found that the miniplate orientation and shape are not the primary factors affecting the stability; the location of the miniplates (superior, middle, or inferior) was determined to be the main parameter by using finite element analysis (FEA) simulation. Champy et al. [9] determined "the ideal line of osteosynthesis in the mandible," where miniplate fixation is the most stable. Therefore, when comparing the stability of BSSO techniques, not only the location for the osteotomy cut but also the location of the miniplate may influence mandibular stability. Therefore, to compare the stability of different lateral osteotomy methods absolutely, we should eliminate the possibility that the location of the miniplates will affect the stability.

FEA is widely used in engineering and can also be used to solve complex problems in dentistry [10]. Several authors have reported the accuracy of FEA for describing the biomechanical behavior of bony specimens [11-13]. We had earlier reported the feasibility of FEA simulation to compare experimental studies and FEA simulations [14]. Vollmer et al. [15] have found quite a high correlation between FEA simulation and *in vitro *measurements of mandibular specimens (correlation coefficient = 0.992). FEA is therefore a suitable numerical method for addressing biomechanical questions and a powerful research tool that can provide precise insight into the complex mechanical behavior of the mandible affected by mechanical loading, which is difficult to assess by other means [16-18].

In this study, we aimed to assess three lateral osteotomy designs (i.e., cuts at the ramus, mandibular angle, and mandibular body regions) from the viewpoint of biomechanical stability and the complex biomechanical behavior of the mandible and screw-miniplate system. For this, we used FEA simulations of three BSSO techniques with miniplate fixation at four different locations, resulting in 12 FEA miniplate fixation methods. We then applied incisal and contralateral molar compressive loads to compare the resultant incisal and bilateral molar displacements as well as the maximum von Mises stress in the screw-miniplate system and maximum bone stress in the vicinity of the screws among the miniplate fixation methods. Here, we show that the Obwegeser-Dal Pont method for BSSO allows the greatest mechanical stability of the mandible.

## Methods

### Mandibular modeling

We performed a computed tomography (CT) scan (Aquillion 64 DAS TSX-1014/HA; Toshiba Medical Systems, Tokyo, Japan) of a synthetic mandible model (8596; Synbone AG, Malans, Switzerland) made of polyurethane. The polyurethane replica was created from exactly matched human anatomy in all dimensions and proportions [19]. A three-dimensional (3-D) FEA model was constructed from 0.5-mm serial axial sections apart from the two-dimensional (2-D) CT image. The model consisted of 134,836 elements and 29,582 nodes. For simplification, bone was assumed to be a single homogenous phase. The material properties were defined as Young's modulus of 13.7 GPa and Poisson's ratio of 0.3 [20]. We then simulated osteotomy on the model by using each of three BSSO techniques. The distal segments were advanced by 5 mm parallel to the occlusal plane without allowing change in the condylar position and thenfixed with bilateral monocortical miniplate fixation using four screws per miniplate. We assumed that all the models had perfect slippage at the bone interfaces. All surgical simulations and analyses were performed with Mechanical Finder version 6.0 (Research Center Computational Mechanics, Tokyo, Japan).

### The BSSO techniques

**Figure 1:**
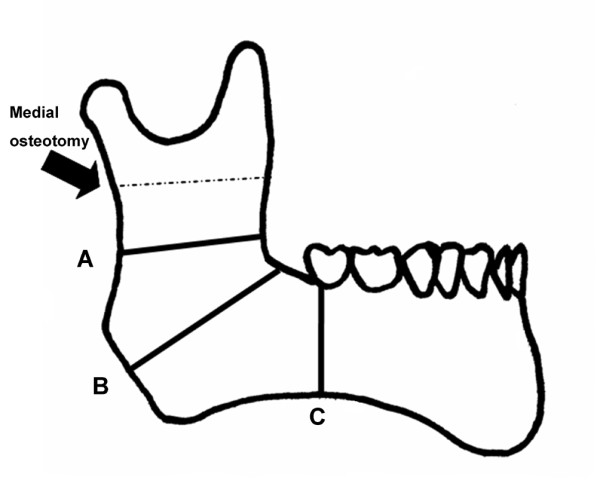
**Schematic of the three lateral osteotomy designs for bilateral sagittal split osteotomy (BSSO)**. (A) In the Trauner-Obwegeser (TO) method, the lateral osteotomy cut was made horizontally from the distal region of the second molar to the posterior border well above the mandibular angle. (B) In the Obwegeser (Ob) method, the lateral osteotomy cut was made from the distal region of the second molar to the midpoint of the mandibular angle. (C) In the Obwegeser-Dal Pont (OD) method, the lateral osteotomy cut was made vertically from the distal of second molar to the lower border of the ascending ramus.

Mandibular biomechanical stability was compared among three BSSO techniques (Fig. 1). In the Trauner-Obwegeser (TO) method, the lateral osteotomy cut was made horizontally from the distal region of the second molar to the posterior border well above the mandibular angle. This osteotomy technique was first performed in 1955 [21] and published in English in 1957 [2].

In the Obwegeser (Ob) method, which was introduced in 1957 [21], the lateral osteotomy cut was made from the distal region of the second molar to the midpoint of the mandibular angle.

In the Obwegeser-Dal Pont (OD) method, the lateral osteotomy cut was made vertically from the distal of second molar to the lower border of the ascending ramus. This osteotomy technique was first performed in 1958 [21] and published in English in 1961 [3].

### Miniplate and screw modeling

Each model was stabilized following the simulated osteotomy by using miniplates and screws. The miniplates were not bent and fit the bone surface as closely as possible. They were simulated as four-hole, straight titanium miniplates (447-224; Synthes Maxillofacial, West Chester, PA) of 1.0-mm thickness by using the 3-D computer-aided design software SolidWorks2008 (SolidWorks Japan, Tokyo, Japan). The screws were simulated as simple 2.0-mm cylinders of length appropriate for monocortical penetration and miniplate fixation. We assumed perfect adaptation between the plate hole and screw through which it was mounted as well as between the screws and bone with no slippage at their interface [8]. The titanium plates and screws were modeled with Young's modulus of 110 GPa and Poisson's ratio of 0.34, using previously reported data [22]. The material properties were the averages of the values in the literature [23,24].

### Miniplate locations

**Figure 2:**
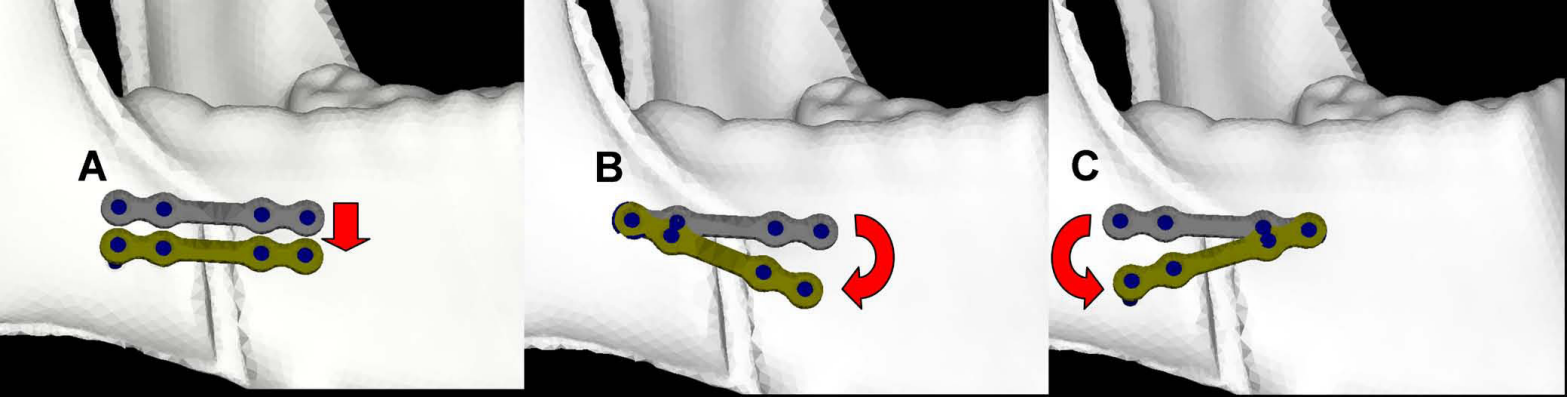
**Miniplate locations**. The baseline location was along Champy's lines; the miniplate was applied along Champy's lines of ideal osteosynthesis as close to the alveolar border as possible (the upper miniplates). (A) The miniplate was placed in translation 5 mm inferior to the baseline location. (B) The miniplate was placed 20° in clockwise rotation to the baseline location. (C) The miniplate was placed 20° in counterclockwise rotation to the baseline location.

**Figure 3:**
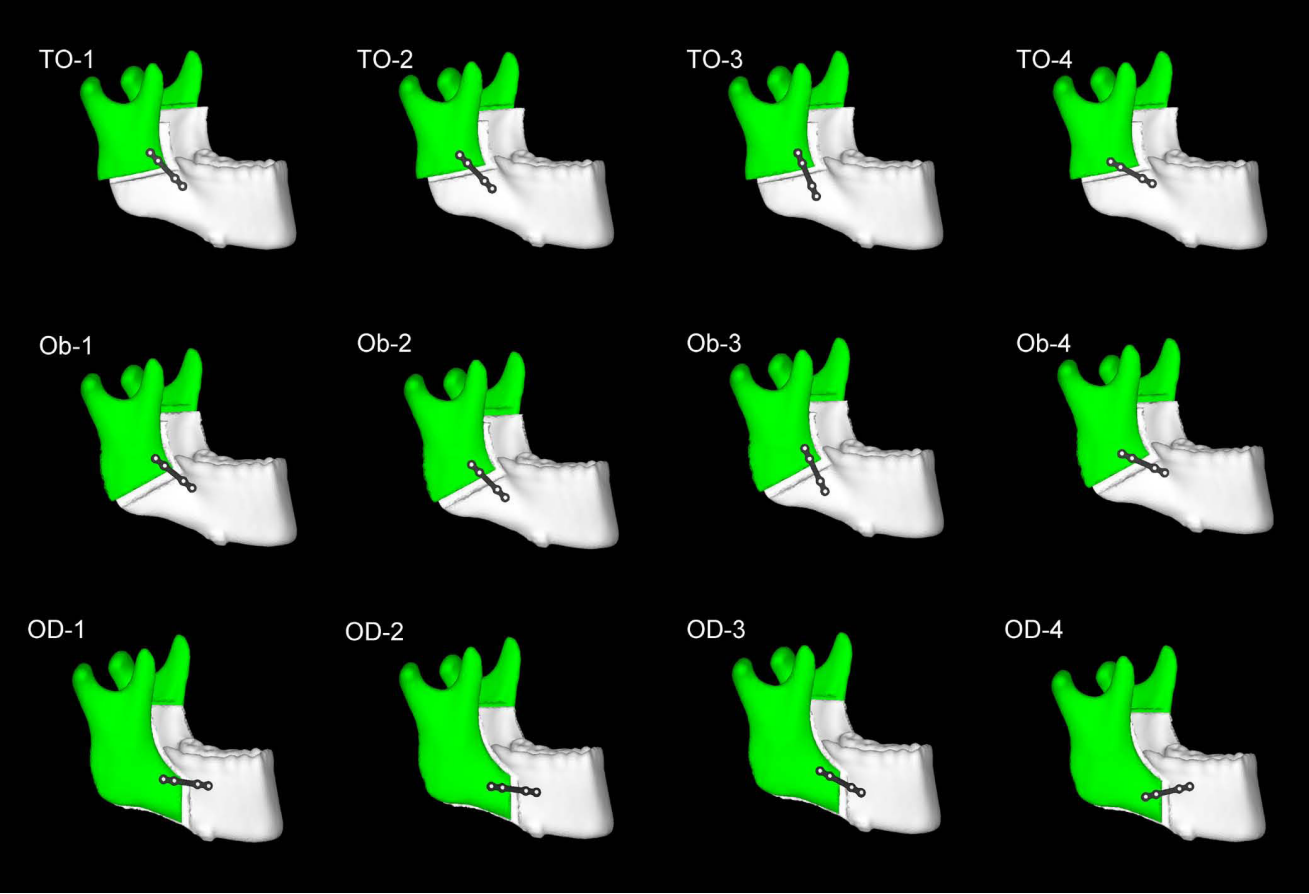
**The 12 finite element analysis (FEA) miniplate fixation models**. TO-1 to 4, the Trauner-Obwegeser method; Ob-1 to 4, the Obwegeser method; OD-1 to 4, the Obwegeser-Dal Pont method. The miniplates were fixed as described in Figure 2.

The three BSSO techniques were divided into four subgroups each. We compared mandibular biomechanical stability among four miniplate locations (Fig. 2), which are frequently encountered inadvertently in the clinical setting. Therefore, 12 different FEA miniplate fixation methods were developed (Fig. 3), as follows:

1. A miniplate was applied along Champy's lines of ideal osteosynthesis, as close to the alveolar border as possible (OD-1, Ob-1, and TO-1 methods).

2. A miniplate was placed in translation 5 mm inferior to the first location (OD-2, Ob-2, and TO-2 methods).

3. A miniplate was placed 20° in clockwise rotation to the first location (OD-3, Ob-3, and TO-3 methods).

4. A miniplate was placed 20° in counterclockwise rotation to the first location (OD-4, Ob-4, and TO-4 methods).

### Constraints

**Figure 4:**
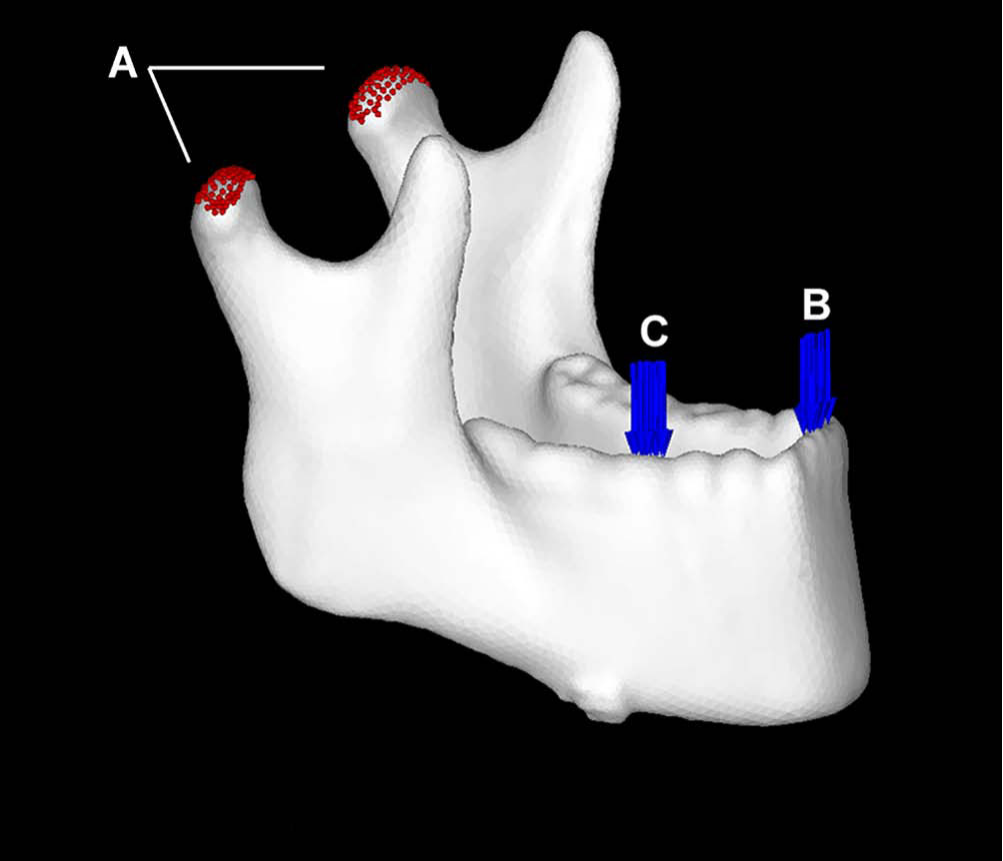
**Establishing the constraints and loading**. (A) The bilateral temporomandibular joints were completely constrained. (B) For incisal loading, a 66.7-N compressive load was applied to the central incisors perpendicular to the occlusal plane. (C) For contralateral molar loading, a 260.8-N compressive load was applied to the occlusal surface of the right first molar perpendicular to the occlusal plane.

The bilateral temporomandibular joints were completely constrained (Fig. 4A).

### Loading

We examined these models under two different loads. For incisal loading, a 66.7-N compressive load was applied to the central incisors perpendicular to the occlusal plane (Fig. 4B). For contralateral molar loading, a 260.8-N compressive load was applied to the occlusal surface of the right first molar perpendicular to the occlusal plane (Fig. 4C).

### The evaluated parameters

For assessing the stability in the three BSSO techniques, central incisor displacement on incisal and contralateral molar loadings, the maximum von Mises stress in the screw-miniplate system, and the maximum bone stress in the vicinity of the screws on both loadings were examined and compared.

For assessing the complex biomechanical behavior on incisal and contralateral molar loadings, first molar displacement bilaterally, the maximum von Mises stress in the bilateral screw-miniplate system, and the maximum bone stress in the vicinity of the bilateral screws in the OD-1, Ob-1, and TO-1 methods were examined. Namely, we compared the working side and balancing side on contralateral molar loading.

## Results

### Central incisor displacement, maximum bone stress, and maximum von Mises stress

Comparisons of the predicted central incisor displacements, maximum predicted bone mechanical stress in the vicinity of the screws, and maximum predicted von Mises stress in the screw-miniplate system on incisal loading and contralateral molar loading are shown in Table 1 and Table 2, respectively. On comparing the three BSSO techniques, the OD method showed the least central incisor displacement, least maximum bone mechanical stress in the screw vicinity, and least von Mises stress in the screw-miniplate system on both loadings, followed by the Ob method and TO method. Similarly, on comparing the four miniplate locations, the Champy's lines models (OD-1, Ob-1, and TO-1 methods) showed the least tooth displacement, least maximum bone stress in the screw vicinity, and least maximum von Mises stress in the screw-miniplate system on both loadings, again followed by the Ob method and TO method.

**Table 1:** Summary of the comparative results for incisal loading

Parameter	Model	TO method	Ob method	OD method
Deflection at the central incisor (mm)	1	5.323	4.180	3.038
	2	5.635	4.550	3.286
	3	6.780	4.235	3.222
	4	6.989	4.661	3.539
Maximum von Mises bone stress in the screw vicinity (MPa)	1	249.981	190.631	110.492
	2	269.497	219.385	132.409
	3	289.571	191.092	131.958
	4	289.737	253.757	139.572
Maximum von Mises stress on the miniplate (MPa)	1	1459.151	1421.798	1124.772
	2	1492.856	1450.541	1247.729
	3	1763.471	1443.686	1216.838
	4	1939.372	1559.816	1289.623
Maximum von Mises stress on the screws (MPa)	1	904.507	827.426	809.941
	2	921.232	919.923	858.749
	3	926.003	854.493	829.947
	4	964.445	983.235	914.539

**Table 2:** Summary of the comparative results for contralateral molar loading

Parameter	Model	TO method	Ob method	OD method
Deflection at the central incisor (mm)	1	11.357	8.522	4.255
	2	14.222	9.665	4.877
	3	15.114	8.630	4.786
	4	15.271	9.931	4.972
Maximum von Mises bone stress in the screw vicinity (MPa)	1	512.634	361.865	256.623
	2	726.506	463.139	325.129
	3	730.439	405.240	320.893
	4	775.176	504.709	356.547
Maximum von Mises stress on the miniplate (MPa)	1	3250.620	2955.626	1766.932
	2	3549.566	3195.330	2082.756
	3	3878.755	3127.397	1941.177
	4	4261.597	3381.705	2156.119
Maximum von Mises stress on the screws (MPa)	1	2118.952	1778.286	1591.128
	2	2239.526	2047.883	1639.485
	3	2336.934	1826.871	1609.271
	4	2397.104	2476.061	1754.459

### Detailed analyses of the Champy's lines model in each BSSO technique

**Figure 5:**
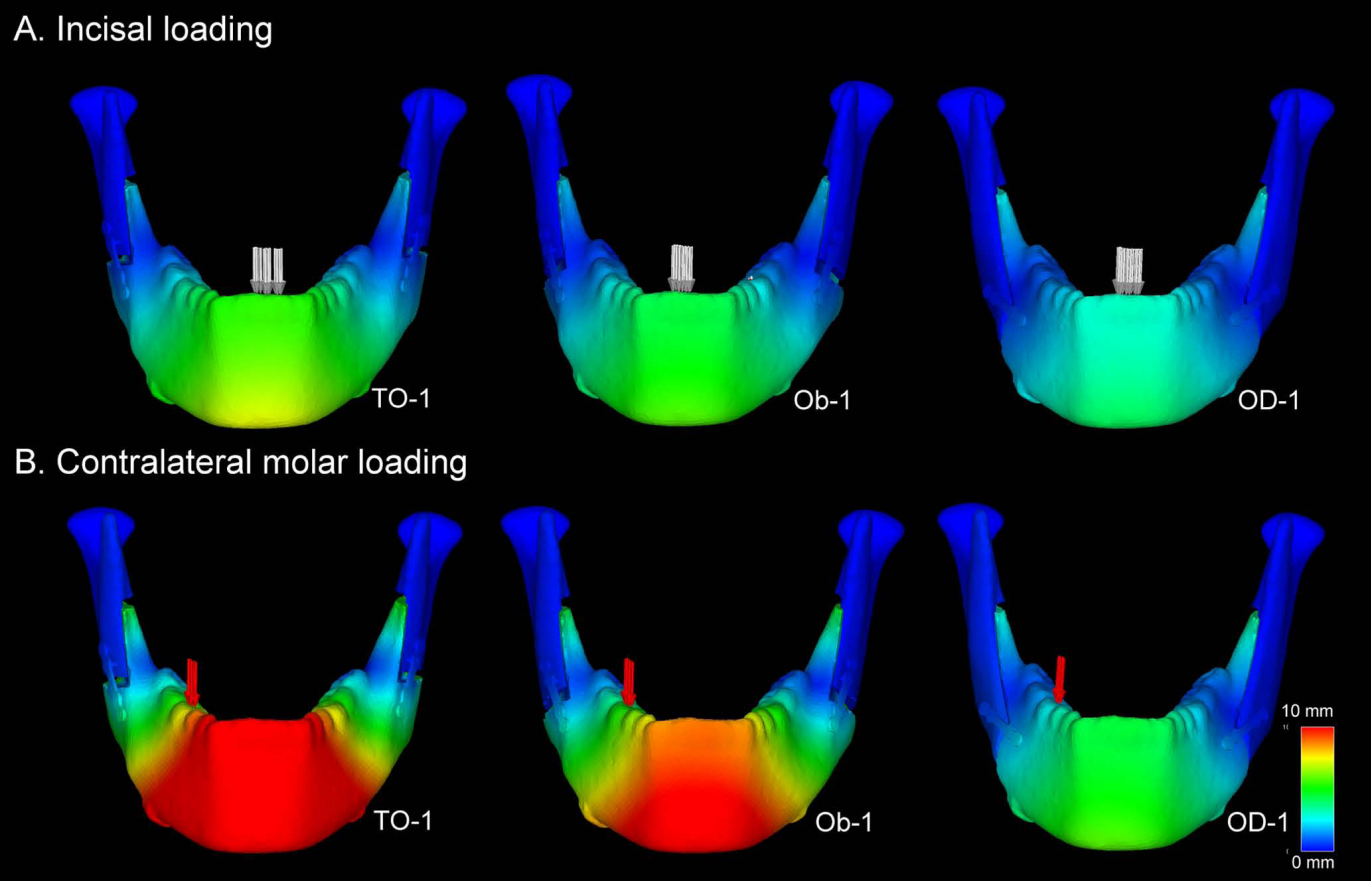
**The displacement fields in the mandibles in the OD-1, Ob-1, and TO-1 methods**. The displacement fields in the mandibles of the Champy's lines models were determined following (A) incisal loading and (B) contralateral molar loading.

**Figure 6:**
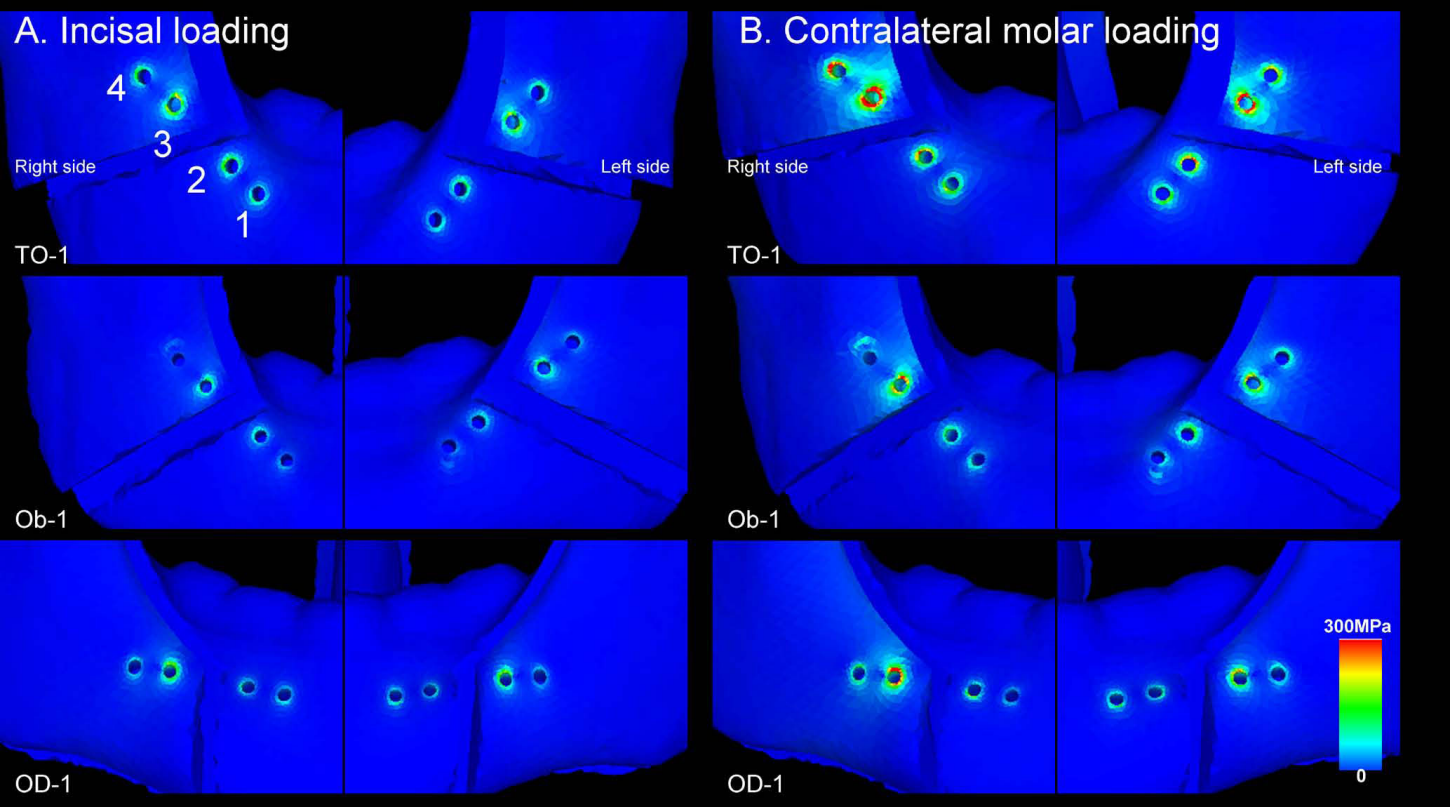
**Regional distributions of von Mises bone stress in the vicinity of the screws in the OD-1, Ob-1, and TO-1 methods**. The highest concentration of bone mechanical stress was found at site 3 bilaterally in all three methods on (A) incisal loading and (B) contralateral molar loading.

**Figure 7:**
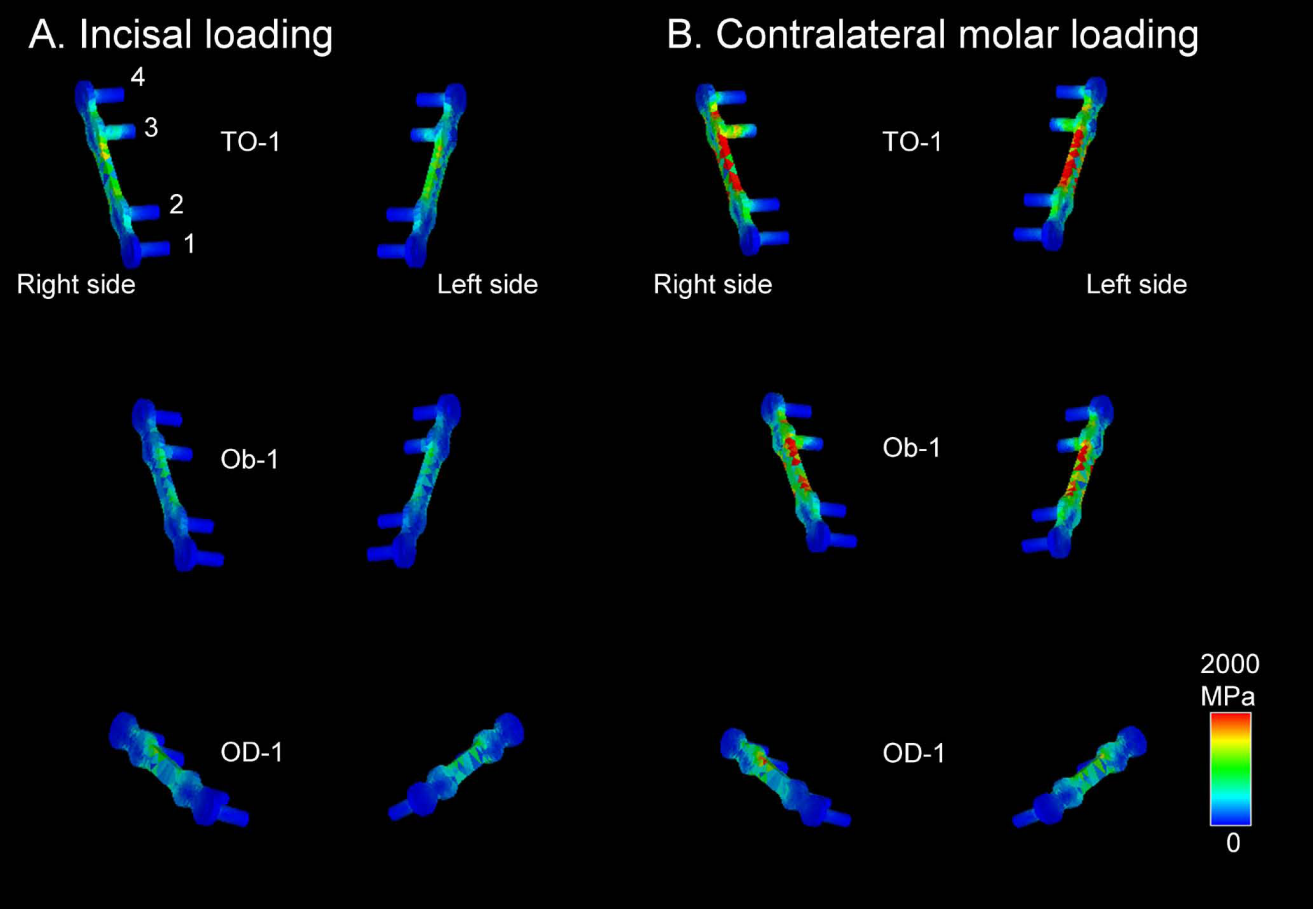
**Regional distributions of von Mises stress on the bilateral screw-miniplate systems in the OD-1, Ob-1, and TO-1 methods**. The site 3 screws and miniplates demonstrated very high tensile stresses in all three methods on (A) incisal loading and (B) contralateral molar loading.

The displacement fields in the mandibles of the Champy's lines models on incisal and contralateral molar loadings are presented in Figure 5. Comparisons of the predicted bilateral first molar displacements, maximum bone mechanical stress in the vicinity of the bilateral screws, and von Mises stress in the bilateral screw-miniplate systems on incisal loading and contralateral molar loading are shown in Table 3 and Table 4, respectively. Regional distributions of von Mises bone stress in the vicinity of the screws and von Mises stress in the bilateral screw-miniplate system of the Champy's lines models on both loadings are shown in Figure 6 and Figure 7, respectively.

**Table 3:** Incisal loading

Parameter	Side	TO-1 method	Ob-1 method	OD-1 method
Deflection at the first molar (mm)	Right	2.786 (100%)	2.068 (100%)	1.231 (100%)
	Left	2.778 (99.7%)	2.053 (99.2%)	1.205 (97.9%)
Maximum von Mises bone stress in the screw vicinity (MPa)	Right	249.981 (100%)	190.631 (100%)	110.492 (100%)
	Left	248.304 (99.3%)	189.818 (99.6%)	101.587 (91.9%)
Maximum von Mises stress on the miniplate (MPa)	Right	1459.191 (100%)	1421.798 (100%)	1124.772 (100%)
	Left	1427.779 (97.8%)	1419.124 (99.8%)	1113.104 (99.0%)
Maximum von Mises stress on the screw (MPa)	Right	904.507 (100%)	827.426 (100%)	809.941 (100%)
	Left	905.978 (100.2%)	823.438 (99.5%)	797.614 (98.5%)

**Table 4:** Contralateral molar loading

Parameter	Side	TO-1 method	Ob-1 method	OD-1 method
Deflection at the first molar (mm)	Right	6.149 (100%)	4.537 (100%)	1.979 (100%)
	Left	5.840 (95.0%)	4.161 (91.7%)	1.708 (86.3%)
Maximum von Mises bone stress in the screw vicinity (MPa)	Right	512.643 (100%)	361.865 (100%)	256.623 (100%)
	Left	441.897 (86.2%)	294.699 (81.4%)	196.790 (76.7%)
Maximum von Mises stress on the miniplate (MPa)	Right	3250.620 (100%)	2955.626 (100%)	1766.932 (100%)
	Left	3101.392 (95.4%)	2598.595 (87.9%)	1665.914 (94.3%)
Maximum von Mises stress on the screw (MPa)	Right	2118.952 (100%)	1778.286 (100%)	1591.128 (100%)
	Left	1964.085 (92.7%)	1663.766 (93.6%)	1474.351 (92.7%)

On incisal loading, for a structurally symmetrical mandible, the bilateral first molar displacements, maximum bone stress, and maximum stress on the screw-miniplate system were nearly symmetrical. In contrast, on contralateral molar loading, the right first molar displacements, maximum bone stress, and maximum stress on the screw-miniplate system were higher than those of the left side.

The screw sites were numbered in all the models as 1-4 from distal (i.e., the ramus) to proximal (i.e., the symphysis) [25]. The highest concentration of bone mechanical stress was found at site 3 bilaterally. Similarly, the site 3 screw and miniplate demonstrated very high tensile stresses.

## Discussion

Using FEA simulation, we have shown that the magnitudes of tooth displacement, the maximum bone stress, and the maximum stress on the screw-miniplate system in the OD method were less than those in the Ob and TO methods at all the miniplate locations on both incisal and contralateral molar loadings. This means that the OD method provided greater resistance to the simulated functional forces than the other two techniques. These results only refer to the miniplate fixation technique and not to screws or semirigid systems.

The smaller size of the lever arm in the OD method probably plays an important role in yielding less stress and smaller displacement. By using FEA simulation, Puricelli et al. [7] suggested that their osteotomy technique presents better mechanical stability than the original OD method. The Puricelli osteotomy is performed at a region further distal to the osteotomy in the OD method, performed nearer to the mental foramen. They speculated that the size of the lever arm decreases as a result of the increased surface area of medullary bone contact [26]; we agree with this interpretation of the results. However, in our FEA simulation, we did not consider bone contact (i.e., all the models were assumed to have perfect slippage at the bone interfaces), because osseous healing starts and is not completed in the early postoperative period. As a matter of course, a larger surface of bone contact promotes faster healing and has less displacement due to muscle activity.

Further, the magnitudes of tooth displacement, the maximum bone stress, and the maximum stress on the plating system were less in the Champy's lines models than in the other models in our study. This means that the Champy's lines models provided greater resistance to the simulated functional forces than the models with other miniplate locations.

Champy and colleagues determined "the ideal line of osteosynthesis" in the mandible, where miniplate fixation is the most stable [27]. In the mandibular angle region, this line indicates that a plate may be placed either along or just below the oblique line of the mandible [9]. Similarly, in our FEA simulation, the models with miniplates placed along Champy's lines demonstrated a trend toward higher stability than those with other miniplate locations. Unfortunately, the ideal sites frequently overlap tooth roots. Avoidance of damage to the roots of teeth and contents of the inferior alveolar canal is important [27].

In an *in vitro *study, Ozden et al. [28] compared the biomechanical stability of ten different fixation methods used in BSSO by using fresh sheep mandibles. Their osteotomy line was similar to that used in our OD method. They tentatively claimed that a miniplate placed obliquely in a clockwise pattern provides greater stability than that placed horizontally. In contrast, in our FEA simulation, the miniplate placed horizontally (OD-1 method) provided greater biomechanical stability than that placed obliquely in a clockwise pattern (OD-3 method). Similarly, in the other BSSO techniques, the rotated miniplate model provided less stability than the Champy's lines models. Therefore, the relationship between angular variation of a miniplate and orientation of the loading may contribute to mechanical stability. However, this relationship has not been systematically studied and warrants further investigation.

Dal Pont et al. [3] demonstrated that the advantages of the OD method are better and easier adaptation of the fragments; broader contact surfaces; greater possibility for correction of prognathism, micrognathia, and apertognathia; and avoidance of as much muscular displacement as possible. On the basis of our findings, we can append another advantage: the OD method provides greater resistance to functional forces than the other BSSO techniques. Good stability of the mandible in the early postoperative period may contribute to primary bone union, immediate postoperative function, and a shortened maxillomandibular fixation period. Moreover, Dolce et al. [29] reported that most of the relapse occurs within the first 8 weeks postsurgically, consistent with the findings of other authors.

Furthermore, when we observed the Champy's lines models closely, the tooth displacements and stresses on the mandible bilaterally were in the same range on incisal loading. In contrast, on contralateral molar loading, the displacements and stresses on the working side were greater that those on the balancing side. The magnitude of all the parameters on the balancing side accounted for about 80% of that on the working side, which is higher than we had thought. Korioth and Hannam [30] have indicated that under conditions of static equilibrium and within the limitations of the current modeling approach, the human jaw deforms elastically during symmetrical and asymmetrical clenching tasks. This deformation is complex, and includes the rotational distortion of the corpora around their axes. In addition, the jaw deforms parasagittally and transversely.

A wide range of magnitudes of chewing forces after BSSO has been reported [31-33]. We assumed the early postoperative condition in this FEA simulation. Masticatory loads of 66.7 N on the central incisors and 260.8 N on the right first molar were simulated, corresponding to the mean immediate postoperative (mandibular advancement) bite force [33]. Although such bite forces were not measured experimentally, it is possible to estimate them by multiplying the rates of improvement [33].

We evaluated the biomechanical behavior in the three BSSO techniques following fixation using miniplates and screws. Although the applied incisal loading mimicked vertically deforming forces and molar loading mimicked torsionally deforming forces encountered under clinical circumstances, they cannot completely represent the complex interaction between the mandible and musculature in function. Therefore, we can only expect to identify trends in behavior that will help in making decisions clinically [34].

In our study, the highest concentration of bone mechanical stress was found at site 3 in all the Champy's lines models. Similarly, the highest concentration of mechanical stress was found on the site 3 screws and upper outer rim of the miniplate near site 3. Chuong et al. [25] produced a 3-D finite element model and examined the stress on fixation after BSSO. They reported that the stress was concentrated on the upper outside rim of the miniplate near site 3, as seen in our results. It has been suggested that this stress concentration is responsible for the screw loosening and miniplate breakage seen clinically [35,36].

Armstrong et al. [37] reported the limitations of *in vitro *experimental study for comparing the multitude of rigid fixation systems. These limitations are almost the same as those of FEA simulation and include the following: the fixation systems were tested by using forces applied vertically, whereas mixed vertical, lateral, and rotational forces may be encountered clinically as dictated by the anatomical environment; the *in situ *plates may be affected by the physiological environment (e.g., inflammation or infection); and the plates were subjected to a single continuous load and not repeatedly loaded as in normal function. In addition to these limitations, FEA simulation also has some inherent limitations [10,16]. The values of the stresses provided by FEA are not necessarily identical to the real ones. In this study, we made several assumptions and simplifications regarding the material properties and model generation. In FEA models, bone is frequently modeled as isotropic, but it is actually anisotropic. In this study, bone was modeled as homogeneous, isotropic, and linearly elastic. Another crucial limitation is that the miniplates were not bent, whereas the plates are often adapted to fit the contour of the bone surface clinically. Nonetheless, the FEA simulation allowed realistic representation of the stress distribution in the fixation material.

## Conclusions

The OD method allows greater mechanical stability of the mandible than the other two BSSO techniques. In addition, miniplates placed along Champy's lines provide greater mechanical advantage than those placed at other locations.

## Competing interests

The authors declare that they have no competing interests.

## Authors' contributions

HF conceived the study design. HT conceptualized the study design, wrote the manuscript, and participated in the FEA analyses. SM, YS, and HM participated in the FEA analyses. TK edited and reviewed the manuscript. All authors read and approved the final manuscript.
